# Rapid means of biofilm disruption induce the newly released (NRel) phenotype of enhanced antibiotic sensitivity

**DOI:** 10.3389/fmicb.2026.1734540

**Published:** 2026-04-10

**Authors:** Joseph Wickham, Kunal R. More, Amber L. Hendricks, Lauren O. Bakaletz, Steven D. Goodman

**Affiliations:** 1 Center for Microbe and Immunity Research, Abigail Wexner Research Institute at Nationwide Children’s Hospital, Columbus, OH, United States; 2 Department of Pediatrics, College of Medicine, The Ohio State University, Columbus, OH, United States

**Keywords:** antibiotic sensitivity, bacterial biofilms, disruption, newly released, *NTHI*

## Abstract

**Introduction:**

Biofilms are communities of microorganisms encased in a self-produced matrix, a structure that makes resident bacteria up to 1,000 times more resistant to antibiotics than their free-living, or planktonic, counterparts. Intriguingly, new methods that use reagents to release the biofilm-resident bacteria result in a transitory yet highly antibiotic sensitive phenotype. These newly released (NRel) bacteria are at least, if not more, sensitive to antibiotics than those in their planktonic form. Here, we sought to determine if the production of the NRel phenotype is reagent- dependent or can be accomplished by alternative means.

**Methods:**

Across four pathogenic bacteria: non-typeable *Haemophilus influenzae (NTHI), methicillin resistant Staphylococcus aureus (MRSA), Pseudomonas aeruginosa*, and *Streptococcus pneumoniae*, we investigated whether rapid mechanical disruption of biofilms, or the use of a novel cationic depletion method in *NTHI*, could similarly induce this NRel phenotype. The presence of NRel was assessed by comparing the antibiotic sensitivity of the released bacteria to that of their planktonic counterparts. For *NTHI* specifically, we further characterized the phenotype by measuring the kinetics of antibiotic sensitivity via comparative plate counts over time. We also analyzed the relative expression levels of known NRel-associated genes using quantitative reverse transcription polymerase chain reaction (qRT-PCR).

**Results:**

We show that using either intense mechanical disruption or a cationic depletion method not only facilitate rapid biofilm disruption but produce the NRel phenotype. In each case, both NRel signature gene expression and the transient antibiotic sensitivity phenotypes were observed compared to planktonic cells.

**Discussion:**

Similar to methods using reagents, we found that mechanical or cationic depletion disruption of pathogenic bacterial biofilms were sufficient to trigger the NRel phenotype. These results are consistent with the NRel state potentially being a rate-dependent physiological response rather than being induced by specific chemical or biological agents. We discuss the changes in gene expression permissive to NRel and the possibility that rapid and premature release of bacteria from a biofilm fails to allow the resident bacteria to be sufficiently prepared for their biofilm free state. This new insight both expands our understanding of the NRel phenotype and provides further validation for our rapid-release therapeutic strategy.

## 1 Introduction

Bacterial biofilms significantly contribute to infection pathogenesis and chronicity (Kolpen et al., 2022). These bacteria reside within a self-produced extracellular polymeric substance (EPS) matrix, which consists of many components, including extracellular DNA (eDNA), proteins, lipids, polysaccharides, and other biopolymers. According to the NIH, up to 80% of human bacterial infections go through a biofilm phase (Fleming and Rumbaugh, 2017), and they play a key role in such common chronic diseases as otitis media, chronic obstructive pulmonary disease, periodontitis, and cystic fibrosis, among others (Costerton et al., 1999; Mirzaei et al., 2020; Vestby et al., 2020). The global economic burden of biofilm-related healthcare costs was estimated to be almost $400 billion in 2019 (Cámara et al., 2022), thus underscoring the need for effective treatment options. However, the biofilm matrix provides a protective barrier against harsh environments, antimicrobials, and host immune effectors (Gunn et al., 2016; Flemming and Wingender, 2010; Koo et al., 2017), often leading to disease recurrence (Lebeaux et al., 2014). While biofilm stability is influenced by species-specific matrix components, a key factor contributing to the structural integrity of mature biofilms is the conversion of DNase sensitive eDNA in the B-form to the DNase-resistant Z-form, which accumulates due to the activity of DNABII proteins (Buzzo et al., 2021). Bacteria in a biofilm state can be 1,000 times more resistant to conventional antibiotic treatments compared to their free living planktonically grown counterparts (Mah, 2012; Sharma et al., 2019; Van Acker et al., 2014). Importantly, new methods are needed to effectively treat these common and highly resistant biofilm-related infections.

A growing number of laboratories are working toward those treatment goals (Kaplan, 2010; Koo et al., 2017; Rumbaugh and Sauer, 2020; Petrova and Sauer, 2016). The approaches these laboratories are taking vary from triggering biofilm dispersal to actively disrupting the biofilm through various means. Dispersal is a bacteria-specific, programmed response to an induced signal, whereby resident bacteria actively escape the biofilm to colonize other sites and start the biofilm life cycle anew (Rumbaugh and Sauer, 2020). Disruption, however, is generally a fast, unprogrammed response to external factors, such as mechanical stress and shearing or reagents that actively target biofilm components to break them apart (Wille and Coenye, 2020). Current methods that use reagents to disrupt biofilms and rapidly release the resident bacteria yield a transient but highly antibiotic-sensitive phenotype wherein the released cells are often more antibiotic sensitive than their planktonic counterparts that seeded the original biofilm (Petrova and Sauer, 2016; Li et al., 2014; Marks et al., 2013; Chambers et al., 2017; Goodwine et al., 2019; Sauer et al., 2004; Zemke et al., 2020).

In previous studies from our group (Mokrzan et al., 2018, 2020; Kurbatfinski et al., 2022; Wilbanks et al., 2023; Kurbatfinski et al., 2023), we have utilized an epitope-targeted monoclonal antibody approach against the DNABII family of DNA binding proteins that serve as linchpins by binding to and converting B-DNA into Z-DNA which act as a structural support for the biofilm matrix. These monoclonal antibodies are directed against the DNA binding tip region of DNABII proteins and take advantage of the natural equilibrium of the DNA bound and unbound state of DNABII proteins. These antibodies in effect sequester the DNABII proteins in the unbound state and prevent their rebinding to the eDNA matrix and thereby shift the equilibrium of the proteins away from the eDNA matrix (Novotny et al., 2016; Goodman and Bakaletz, 2022). The induced equilibrium shift of the DNABII proteins out of the matrix results in a rapid collapse (within minutes) of the biofilm and release of the resident bacteria. These newly released (NRel) bacteria display a transient yet significantly increased antibiotic susceptibility (Kurbatfinski et al., 2022, 2023; Mokrzan et al., 2020; Wilbanks et al., 2023), and a unique gene expression profile (Mokrzan et al., 2020).

Many of these disruption methods are rapid in nature, occurring in the span of minutes (Mokrzan et al., 2020; Kalia and Sauer, 2024; Koo et al., 2017; Guilhen et al., 2017; Kaplan, 2010; Petrova and Sauer, 2016; Chua et al., 2014; Chambers et al., 2017; Whitchurch et al., 2002; Kaplan et al., 2012). According to our hypothesis, the rate of disruption is essential to produce the NRel phenotype so as to yield released bacteria that are ill-prepared via apropos gene expression to sufficiently protect themselves against the newly encountered harsh environment in the absence of the biofilm. Therefore, it stands to reason that the kinetics of the disruption would matter. To that end, and as a proof of concept, we sought to determine if NRel can be produced by other methods of similar kinetics, specifically rapid physical or mechanical means. This is important in that it would allow us to determine if the production of the NRel phenotype is not so much reagent dependent, but rather a result of the rapid nature of the disruption.

As we have demonstrated previously (Kurbatfinski et al., 2022, 2023; Mokrzan et al., 2020; Wilbanks et al., 2023), the NRel phenotype generally consists of three main pillars: increased antibiotic sensitivity, a transient kinetic profile, and a unique gene expression profile. Here, we evaluated four predominant respiratory pathogens [non-typeable *Haemophilus influenzae* (*NTHI*), methicillin resistant *Staphylococcus aureus* (*MRSA*)*, Pseudomonas aeruginosa, and Streptococcus pneumoniae*] using several reagent free and rapid disruption methods to determine if we could induce the NRel state and its three main hallmarks.

## 2 Materials and methods

### 2.1 Bacterial strains and growth

*NTHI* strain 86-028NP (Sirakova et al., 1994) was maintained frozen in LN2 at passage #4 on artificial medium since its original isolation from the nasopharynx of a deidentified child who underwent routine tympanostomy tube insertion due to chronic otitis media (Kurbatfinski et al., 2022). *NTHI* 86-028NP was grown in Brain Heart Infusion broth supplemented (sBHI) with hemin (2 μg/mL) (Sigma-Aldrich, St. Louis, MO; Cat no. H9039) and β-NAD (2 μg/mL) (Sigma-Aldrich, St. Louis, MOl Cat no. N1511) or Chocolate agar. *MRSA* [a clinical isolate from a child with cystic fibrosis (Kurbatfinski et al., 2023)] and *P. aeruginosa* strain 142-1 [an isolate from an ICU patient with nosocomial pneumonia, kindly provided by William Weiss, University of North Texas Health Sciences Center (Kurbatfinski et al., 2022)] were grown on Tryptic Soy agar (TSA) or in Tryptic Soy broth. *S. pneumoniae* 1121 [derived from an isolate of a child with otitis media, kindly provided by Jeffrey Weiser, New York University (Kurbatfinski et al., 2022)], was grown in Todd Hewitt Broth supplemented with 0.2% yeast extract or on Blood agar (TSA with 5% sheep’s blood). All organisms were grown at 37 °C in a humidified atmosphere with 5% CO_2_ for approximately 16 hours.

### 2.2 Antibiotics and determination of antibiotic concentration

Three antibiotics for each of the four respiratory tract pathogens listed above were selected for use in this study based on their clinical relevance, with a preference toward those that would be prescribed for a current infection of the respective bacteria, as well as to bridge from our previous work (Kurbatfinski et al., 2022, 2023) whereby we used the same antibiotics tested with our DNABII monoclonal antibody disruption approach. Minimum inhibitory concentrations (MIC) for each antibiotic as shown previously (Kurbatfinski et al., 2022) were used as a starting point to determine the antibiotic concentration where the percent killing of planktonically grown cultures of each tested bacterium was between ∼15% and 25% so as to be able to demonstrate any enhanced sensitivity to killing by NRel bacteria. The organisms, antibiotics used, and their respective working concentrations are shown in Table 1.

**TABLE 1:** Organisms and antibiotics used in this study.

Organism tested	Antibiotics used	Working concentrations (μg/mL)
*NTHI* 86-028NP	Trimethoprim + sulfamethoxazole*	0.125
	Amoxicillin + clavulanate	0.5
	Clarithromycin	1.0
*MRSA*	Vancomycin	0.05
	Levofloxacin	0.005
	Linezolid	0.1
*P. aeruginosa* 142-1	Ceftazidime	4.0
	Tobramycin	2.0
	Piperacillin	2.0
*S. pneumoniae* 1121	Vancomycin	0.05
	Amoxicillin + clavulanate	0.003
	Trimethoprim + sulfamethoxazole*	4.0

*Trimethoprim-sulfamethoxazole was prepared in a 1:19 ratio according to EUCAST guidelines as described previously (Kurbatfinski et al., 2022). The concentration value for trimethoprim-sulfamethoxazole is expressed as the concentration (μg/mL) of trimethoprim.

### 2.3 Mechanical disruption

*NTHI* was grown as described above in sBHI for 16 h at 37 °C with 5% CO_2._ The CFUs (colony forming units) released from these *NTHI* biofilms using a combination of sonication for 0–5 min, vortexing (Fisher analog vortex, Cat # 02215414, Model 9454FIALUS) at speed setting 10 with and without 6 mm borosilicate glass beads coated with SigmaCote™ (Sigma-Aldrich, St. Louis, MO) for up to 5 min and pipetting (Thermo Scientific™, Waltham, MA Finnpipette™ F2 20–200 μL model, Fisherbrand Redi-Tip™ Cat 02707501) multiple times for up to 5 min followed by serial dilutions and plating on chocolate agar was evaluated. Pipetting and sonicating as well as vortexing with beads consistently released the highest number of viable CFUs.

These methods were then utilized on biofilms of each organism grown for 16 h at 37 °C under 5% CO_2_ in 48-well plates. These 16 h biofilms were then gently washed two times with PBS (except for *S. pneumoniae* which was washed only once due to its more delicate biofilm structure), then either pipetted up and down for 2 min followed by sonication for 2 min in a Fisher Scientific (Pittsburgh, PA) waterbath sonicator, Model FS20, or scraped from the wells, transferred to Eppendorf tubes that contained siliconized glass beads and vortexed at the highest setting for 2 min (except for *P. aeruginosa*, which was vortexed for 5 min due to its more robust biofilm structure). For planktonic cultures, 6 mL of sBHI was seeded with approximately six individual colonies of freshly plated *NTHI* to achieve an OD_490*nm*_ of 0.1, and allowed to grow at 37 °C, 5% CO_2_ until mid-log phase (2–3 h), before being aliquoted and subjected to the same disruption methods as listed above for biofilm cells.

For mechanically disrupted biofilm biomass reduction experiments, biofilms were first grown in an eight well chamber slide for 16 h. Biofilms were scraped from the chamber slides and pipetted as a suspension before mechanical NRel treatments. The residual biofilm was washed, stained with LIVE/DEAD^®^ stain and visualized using CLSM. COMSTAT software was used for biomass quantification (Heydorn et al., 2000) and then the average biomass against control were plotted using GraphPad Prism (version 10.3.1)

### 2.4 P11 phosphocellulose activation and biofilm assays

A total of 200 mg P11 phosphocellulose resin (Whatman-Cytiva, Marlborough, MA) was washed first in 10 mL Milli-Q water, which was then removed by centrifugation at 500 rpm for 5 min, and the resin was then incubated with 10 mL 0.5 N NaOH (10 min). This treated resin was then washed with Milli-Q water once and then incubated with 10 mL 0.5 N HCl (10 min). Resin was then washed with Milli-Q water and equilibrated in 10 mL of 20 mM sodium phosphate buffer. Before use, the buffer was replaced with media to achieve a final 1.5% w/v P11 resin.

*NTHI* biofilms were initiated in the basolateral chamber of the transwell system (Corning Costar, Part no. 3470), for 24 h, and then P11 or sBHI (300 μL) was added to the apical chamber (1.5% w/v) for 2 h. This treatment disrupts pre-formed biofilms and releases NRel into the media within the basolateral chamber. The media from the basal chamber were then collected carefully without disrupting any remaining biofilms and centrifuged at 10,000 *g* for 10 min to collect NRel. NRel were then washed once with 1 mL PBS then used to assess the antibiotic sensitivity, time course experiments, and RNA isolation as mentioned below. Post-treatment, biofilms in the basolateral chamber were stained with LIVE/DEAD^®^ stain, fixed, and visualized using CLSM. COMSTAT software was used for biomass and thickness quantification (Heydorn et al., 2000) and then the average thickness and biomass against control were plotted using GraphPad Prism (version 10.3.1).

### 2.5 Antibiotic sensitivity assay

**FIGURE 1:**
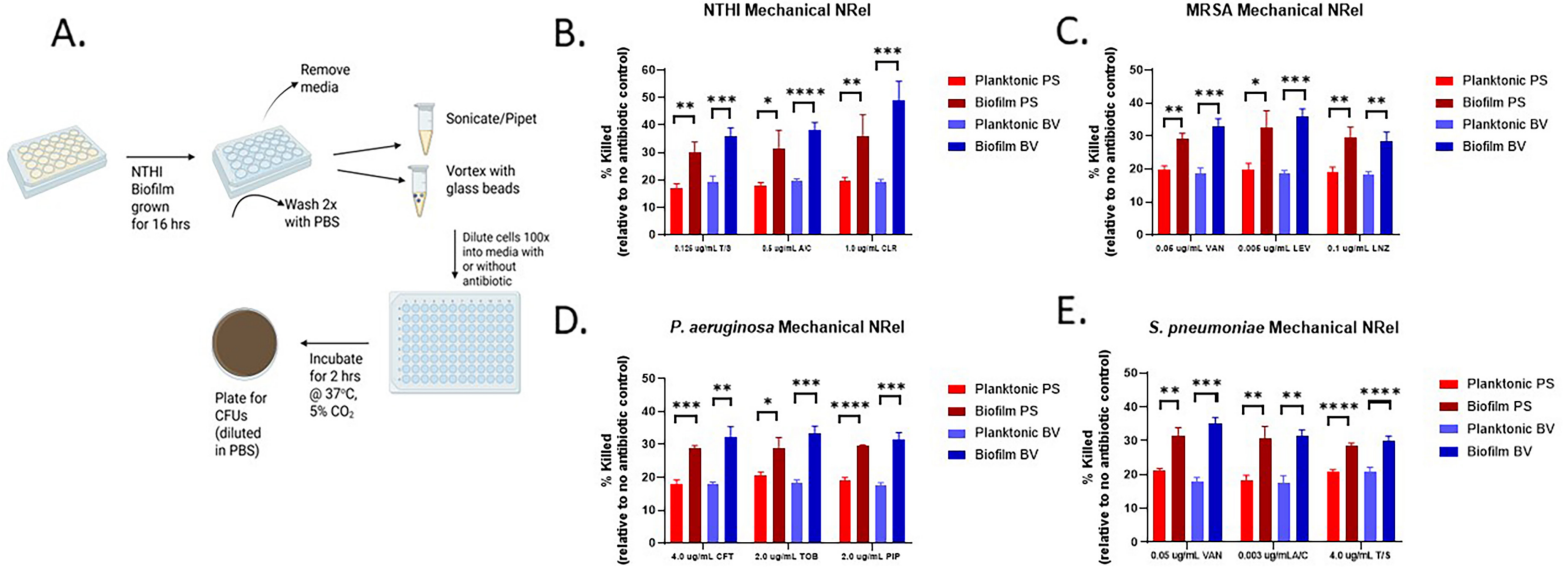
Enhanced antibiotic sensitivity of multiple pathogens after mechanical disruption. **(A)** A schematic which shows the steps taken in the mechanical disruption process. Created in BioRender. Wickham, J. (2026) https://BioRender.com/90ydlt2. **(B)** Antibiotic sensitivity profile of *NTHI* comparing planktonic versus cells released from disrupted biofilms for Trimethoprim + Sulfamethoxazole (T/S), Amoxicillin + Clavulanate (A/C) or clarithromycin (CLR). **(C)** Antibiotic sensitivity profile of *MRSA* comparing planktonic versus cells released from disrupted biofilms for vancomycin (VAN), levofloxacin (LEV), or linezolid (LNZ). **(D)** Antibiotic sensitivity profile of *P. aeruginosa* comparing planktonic versus cells released from disrupted biofilms for ceftazidime (CFT), tobramycin (TOB), or piperacillin (PIP). **(E)** Antibiotic sensitivity profile of *S. pneumoniae* comparing planktonic versus disrupted biofilm cells for vancomycin (VAN), Amoxicillin + Clavulanate (A/C) or Trimethoprim + Sulfamethoxazole (T/S). **(B–E)** Antibiotic sensitivity measured via comparative plate counts as percent killing as mediated by each antibiotic against planktonic or disrupted biofilm cells, the latter of which was achieved by either pipetting and sonication (PS) or vortexing with glass beads (BV). Statistics determined via unpaired *t*-tests. *p ≤ 0.05; **p ≤ 0.01; ***p ≤ 0.001; ****p ≤ 0.0001.

After determining the appropriate antibiotic concentration to achieve ∼15%–25% planktonic killing for each organism and disrupting the biofilms or planktonic cells, as described above, the resulting cell suspensions were then diluted 100-fold into the appropriate media with or without antibiotics and allowed to incubate at 37 °C with 5% CO_2_ for 2 h. This was followed by serial dilutions in PBS and plating for CFUs to determine the relative killing by antibiotics compared to the no antibiotic sample for both planktonic cells and cells released from disrupted biofilms (see schematic Figure 1A).

### 2.6 Time course experiment

To determine the kinetic profile of antibiotic sensitivity (i.e., when sensitivity develops and how long this phenotype endures), we narrowed our focus to *NTHI* using the vortexing with glass beads disruption method. This generally followed the procedure outlined above for the antibiotic sensitivity assay, except that planktonic cells and cells released from disrupted biofilms were incubated with or without amoxicillin plus clavulanate (A/C) or Trimethoprim plus sulfamethoxazole (T/S) for 5, 15 min, 1, 2, 4, and 6 h. At each time point, the cells were diluted and plated as described above to determine comparative plate counts and relative antibiotic sensitivity over time.

### 2.7 RNA isolation and qRT-PCR

For NRel RNA isolation, 6 ml of sBHI was seeded with *NTHI* to achieve 2 × 10^5^ CFU/ml into a 25 cm^2^ cell culture flask (Corning, Corning, NY). The flasks were incubated at 37 °C, 5% CO_2_ for 16 h, then gently inverted, and the medium decanted. The biofilms were washed by carefully adding 2 mL PBS and gently inverting the flask, then inverted again to remove the PBS followed by repeating for a second wash. One mL PBS was added to the flask and the biofilms were gently scraped and pipetted to remove the bacterial cells. The bacterial cell suspension was added to a 1.5 mL Eppendorf tube and centrifuged for 5 min at 4,000 × *g* to pellet the cells. The supernatant was removed, and the pellets were resuspended in 500 μL of PBS and transferred to new tubes that contained siliconized glass beads and vortexed for 2 min. The resulting NRel were removed to a new tube, 500 μL of pre-warmed sBHI added and incubated at 37 °C, 5% CO_2_ for 15 min. The cells were then centrifuged for 1 min at 15,000 × *g*, the supernatant aspirated, and 1 ml TRIzol™ Reagent (ThermoFisher, Waltham, MA) was immediately added to the bacterial pellet. Samples were stored at −80 °C.

For planktonic RNA isolation, 6 mL of sBHI was seeded with *NTHI* to achieve an OD_490_ nm of 0.1, and allowed to grow at 37 °C, 5% CO_2_ until mid-log phase (2–3 h), then centrifuged for 1 min at 15,000 × *g* and the supernatant aspirated. The resulting cell pellets were washed with 1 mL PBS and recentrifuged for 1 min at 15,000 × *g*, the supernatant aspirated, and 1 ml TRIzol™ Reagent was immediately added to the bacterial pellet. Samples were stored at −80 °C.

RNA was purified with a Qiagen RNeasy kit (Qiagen, Germantown, MD) using the optional DNase treatment to remove residual DNA, per manufacturer’s instructions. Anti-DNABII RNA was obtained, as described and used previously in Mokrzan et al. (2020) and Wilbanks et al. (2023). RNA integrity was confirmed through the Qubit RNA IQ assay (Qubit 4 Fluorometer, Invitrogen). RNA was only used downstream if it had a score of 9.0 or higher (out of 10.0). Quantitative reverse transcription-PCR (qRT-PCR) was used to measure relative gene expression via the Superscript III Platinum SYBR Green One-Step qRT-PCR kit (ThermoFisher, Waltham, MA r) per manufacturer’s instructions. Gene expression was normalized to 16S, and relative expression was calculated by the comparative (ΔΔCT) method, with fold change in gene expression expressed as 2^–ΔΔCT^. Results represent the mean of three independent biofilm preparations on separate days, each assayed in duplicate. The primers used (see Supplementary Table 1) were previously utilized in publications from our group (Mokrzan et al., 2020; Wilbanks et al., 2023) and were confirmed in this study by melt curve analysis, indicating specific amplification (e.g., single peaks) for each primer across each RNA tested.

## 3 Results

### 3.1 Determination of mechanical disruption methods

To test our hypothesis that rapid biofilm disruption can produce the NRel phenotype, we first needed to identify the mechanical methods that could best disrupt biofilms while producing the greatest amount of viable, released cells. As a proof of concept, we wanted to evaluate disruption methods that were very rapid in nature and could be executed in the span of minutes, to emulate other rapid, reagent-based methods. As such, *NTHI* was grown for 16 h at 37 °C, 5% CO_2_ and the resulting 16 h biofilms were washed and subjected to combinations of water bath sonication for up to 5 min, vortexing with and without glass beads for up to 5 min, or multiple triturating with a pipet for variable durations to evaluate the viability of the resulting, released cells. Triturating and sonicating for 2 or 5 min each, as well as vortexing with beads for 0.5–5 min consistently released the greatest number of CFUs (Supplementary Figure 1A). To maintain consistency and keep the disruption time short, we opted to utilize the following methods of mechanical disruption in downstream experiments: triturating vigorously for 2 min followed by sonication for 2 min or scraping up the biofilm and vortexing with glass beads for 2 min. To determine the effectiveness of disruption (i.e., were individual cells or cell aggregates being released), we stained the cells released from disrupted biofilms with crystal violet and compared them to planktonic *NTHI* viewed under light microscopy. Both the pipetting and sonication as well as the vortexing with glass beads methodologies were able to yield mostly small cell aggregates, with few individual cells, visually different from unaggregated planktonically grown *NTHI* (Supplementary Figure 1B). The cell aggregation observed here in disrupted preparations is similar to those seen with anti-DNABII exposure and is another phenotype of NRel, as previously reported (Mokrzan et al., 2020). In addition to the visual inspection of NRel via crystal violet staining (Supplementary Figure 1B), the effectiveness of physical biofilm removal from the chamber slide was also determined and shown in Supplementary Figure 2; the scraping process removed >99% of the original biofilm as judged by the reduction in biomass. Finally, we wanted to ensure that the mechanical disruption itself did not affect cell viability. To that end, we tested subsets of planktonic *NTHI* cells with and without beads and vortexing for 2 min at low and high settings (settings 3 and 10 using the Fisher analog vortex mentioned above) to determine relative number of viable bacteria (Supplementary Figure 3). Neither the presence of beads nor the relative forces applied by vortexing demonstrated a significant difference in viability as measured by plate counts for CFUs of the planktonic suspensions, indicating that this disruption method did not result in cell death in and of itself.

### 3.2 Increased antibiotic sensitivity of cells released from mechanically disrupted biofilms

First, and perhaps most important, among the hallmarks of the NRel phenotype is the increased antibiotic sensitivity compared to planktonically grown cells. Here, we sought to determine if our mechanically disrupted biofilm cells displayed increased antibiotic sensitivity. Using the antibiotics outlined in Table 1 and the disruption methods described above, we tested both planktonically grown cells and disrupted biofilm cells of each organism in the presence or absence of antibiotic which were then serially diluted and plated for CFUs (Figure 1A). The percentage of cells killed was determined by comparative plate counts of each cell type with and without antibiotics. Increased % killing of the cells released from disrupted biofilms compared to planktonically grown cells would likely indicate the production of the NRel phenotype. Remarkably, both pipetting/sonication and vortexing with beads yielded bacterial populations that were significantly more sensitive to antibiotic killing compared to their planktonic counterparts for all four tested pathogens (Figures 1B–E). This demonstrates that the increased antibiotic sensitivity characteristic of the NRel phenotype was produced using both methods, albeit to a somewhat greater extent when vortexing with glass beads.

### 3.3 Phosphocellulose (P11)-mediated sequestration of positively charged biomolecules promotes disruption of established biofilms with generation of NRels

As a second approach, we wanted to evaluate a non-mechanical but still rapid form of physical disruption of an *NTHI* biofilm. Electrostatic interactions between negatively charged DNA and positively charged DNABII proteins (e.g., HU and IHF) help stabilize DNA structures within the biofilm matrix (Guéroult et al., 2012; Devaraj et al., 2018). Previous studies have shown that depletion of positively charged molecules such as Mg^++^ from biofilms increases the susceptibility of *NTHI* to antibiotic treatment (Cavaliere et al., 2014), while targeted removal of DNABII proteins from the biofilm matrix due to the action of specific antibodies results in rapid, significant biofilm collapse with concomitant release of NRels (Devaraj et al., 2015; Novotny et al., 2013; Gunn et al., 2016; Rocco et al., 2018; Mokrzan et al., 2020). Ergo, we hypothesized that removal of positively charged molecules may rapidly destabilize the biofilms, to release NRels. To test that hypothesis, we employed phosphocellulose (P11), a well-established cation exchanger and DNA mimetic historically used to purify DNABII proteins (Nash and Robertson, 1981; Vorgias and Wilson, 1991).

**FIGURE 2:**
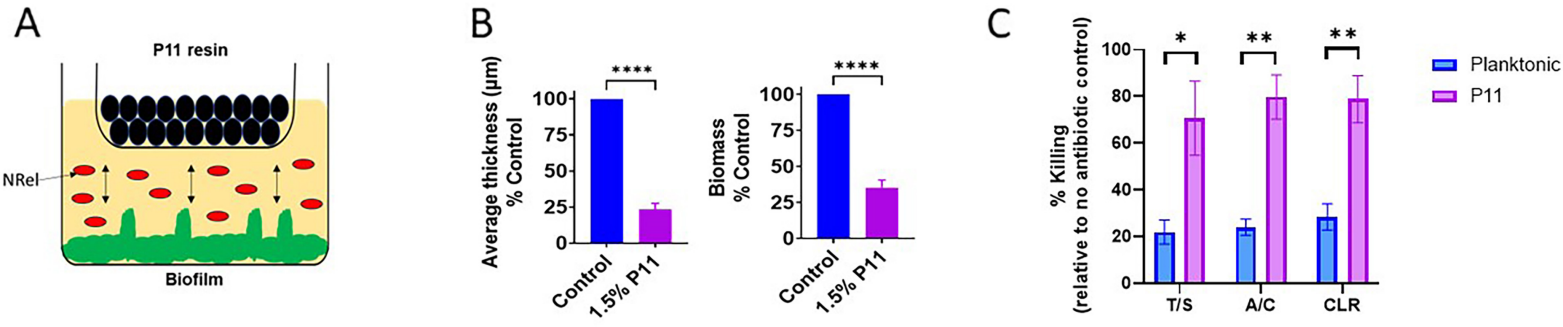
Enhanced antibiotic sensitivity of *NTHI* after P11 treatment of the biofilm. **(A)** A schematic showing how the Phosphocellulose P11 resin works in the transwell setting. Created in BioRender. Wickham, J. (2026) https://BioRender.com/y7b6j6b. **(B)** Disruption of biofilm represented by the reduction in Average thickness and biomass. *NTHI* biofilms were allowed to grow in the basal chamber of the transwell for 24 h, then were co-cultured with 1.5% w/v P11 or sBHI media control within an apical chamber for 2 h. The biofilms were stained with Live/Dead stain, fixed, and visualized using CLSM. The average thickness and biomass were calculated using COMSTAT software and plotted as % control. The data are presented as mean ± SEM of *n* = 4 independent experiments. **(C)** Antibiotic sensitivity profile of *NTHI* comparing planktonic versus cells released from P11 disrupted biofilms for sensitivity to Trimethoprim + Sulfamethoxazole (T/S), Amoxicillin + Clavulanate (A/C) or clarithromycin (CLR). Statistical significance was determined via unpaired *t*-tests. *p ≤ 0.05 **p ≤ 0.01; ****p ≤ 0.0001.

In this approach, we utilized a transwell system in which *NTHI* biofilms were allowed to establish in the basolateral chamber, whereas P11 was added to the apical chamber with the two chambers separated by a 0.4 μm pore membrane (Figure 2A). This setup would theoretically create an equilibrium shift through diffusion and accumulation of small molecules, such as DNABII proteins and other small positively charged species into the apical chamber, while preventing the similar passage of bacterial cells. The apical chamber was supplemented with 0% (w/v) P11 (media control) or 1.5% (w/v) P11, all in sBHI, then incubated for 2 h and analyzed for biomass and average thickness. As shown in Figure 2B, P11 significantly reduced biofilm thickness and biomass, which confirmed biofilm disruption capability. In parallel assays, we collected NRel cells from the basolateral chamber, and comparative plate counts were evaluated in the presence or absence of antibiotics as described above. For consistency, the same antibiotics and antibiotic concentrations that yielded 15%–25% killing of planktonic *NTHI* were used, as listed in Table 1. We observed a significant increase in killing of the cells released from P11 disrupted biofilms compared to planktonic (Figure 2C). Remarkably, P11 disruption resulted in even greater released bacterial killing percentages than mechanical disruption in *NTHI* (Figure 1B). Altogether, three different methods of rapid physical disruption, albeit to different extents, were able to induce the increased antibiotic sensitivity phenotype seen in NRel.

### 3.4 Kinetic profile of antibiotic sensitivity of *NTHI* NRel generated by rapid disruption

**FIGURE 3:**
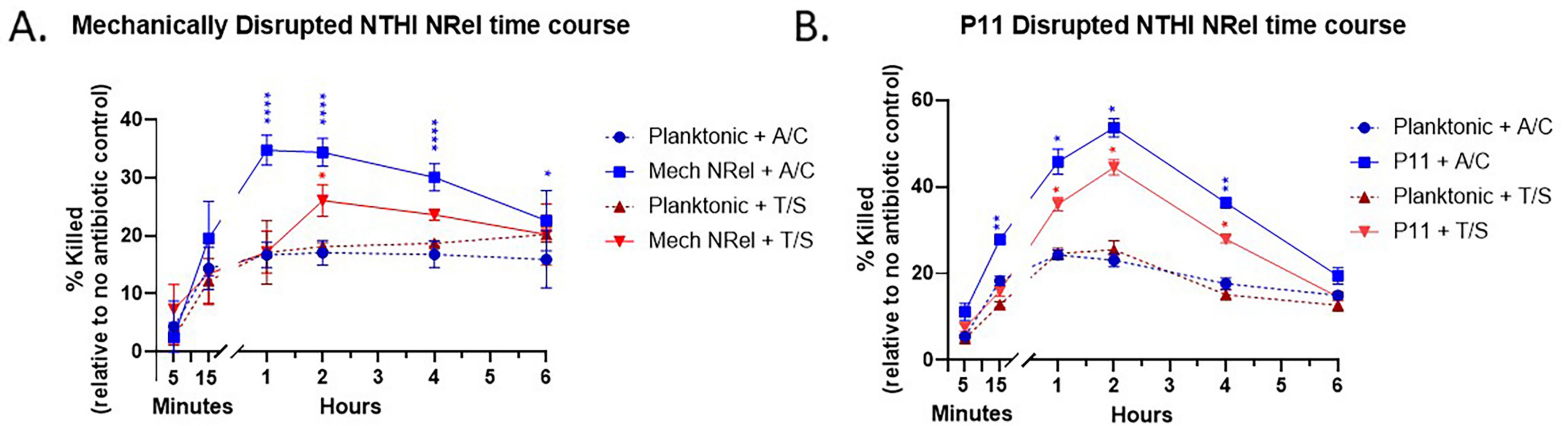
Antibiotic susceptibility of *NTHI* NRel over time. **(A)** Kinetic profile of mechanical *NTHI* NRel sensitivity to A/C and T/S over time. When treated with A/C, NRel started to differentiate from planktonic in terms of relative sensitivity to killing by 15 min, peaked at 1 h and slowly fell back to near planktonic levels by 6 h. When treated with T/S, sensitivity did not reach significance until 1 h, before trending back to planktonic levels. **(B)** Kinetic profile of NTHI NRel generated by co-incubation with P11 sensitivity to killing by A/C and T/S over time. When treated with A/C, P11-generated NRel differentiated from planktonic in terms of relative sensitivity to killing that was significant by 15 min and 4 h, peaking at 2 h and before falling back to near planktonic levels by 4 and 6 h. When treated with T/S, relative sensitivity to killing reached significance between 1 and 4 h, before trending back to planktonic level by 6 h. Statistical significance was determined via two-way ANOVA with multiple comparisons. *p ≤ 0.05; **p ≤ 0.01; ****p ≤ 0.0001.

We next wanted to determine both when enhanced antibiotic sensitivity was first detectable and for how long it persisted, as a measure of the duration of the NRel state post disruption. We narrowed our focus to *NTHI* with either mechanical disruption (e.g., vortexing with glass beads) or via co-incubation with P11, as shown previously, with the exception that planktonic cells and cells released from disrupted biofilms were incubated with or without the antibiotics A/C or T/S for 5, 15 min, 1, 2, 4, and 6 h. At each time point, the cells were diluted and plated to determine comparative plate counts. For our mechanical method, the increased susceptibility to A/C of the cells released from disrupted biofilms started to differentiate from planktonic at 15 min, reached significance that was maintained from 1 to 4 h, then started to return to near planktonic levels by 6 h. Interestingly, mechanically disrupted *NTHI* had a much more muted response to T/S, wherein relative killing was only significantly greater than that observed for planktonically grown *NTHI* at the 2-h time point before falling back to near planktonic levels at 4 and 6 h (Figure 3A). Upon disrupting the biofilms with the P11 resin, the increased susceptibility to A/C of the cells released from disrupted biofilms significantly differentiated from planktonic sensitivity between 15 min and 4 h, peaking at 2 h and falling back to near planktonic levels by 4 and 6 h. When NRel resulting from co-incubation with P11 were treated with T/S, increased sensitivity to killing reached significance between 1 and 4 h, before trending back to planktonic levels at 6 h (Figure 3B).

### 3.5 Relative gene expression in *NTHI* NRel

**FIGURE 4:**
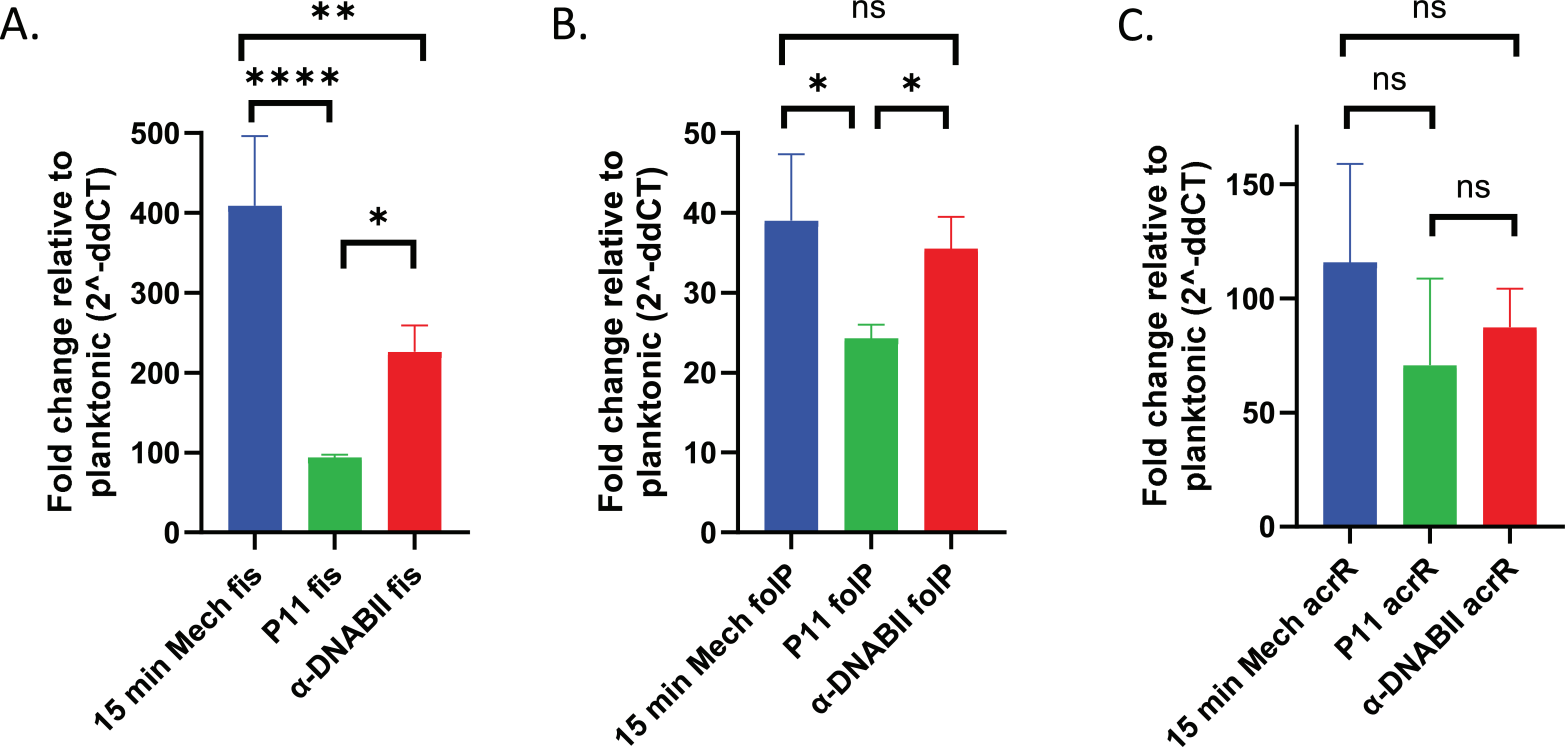
Relative expression of genes canonically associated with lag phase in *NTHI* NRel generated by either mechanical disruption or co-incubation with P11. Relative gene expression profiles for targeted genes in cells released from disrupted *NTHI* biofilms compared to planktonic cells. mRNA was isolated post disruption and analyzed via qRT-PCR with results expressed as fold change relative to planktonic expression (2^– ddCT^). Results represent testing in duplicate across four different days. **(A)** *fis* gene expression profiles in NRel resulting from each of three disruption methods: mechanical, co-incubation with P11 and action of anti-DNABII. **(B)** *acrR* gene expression profiles in NRel resulting from each of three disruption methods. **(C)** *folP* gene expression profiles in NRel resulting from each of three disruption methods. Statistical significance was determined via unpaired *t*-tests. *p ≤ 0.05; **p ≤ 0.01; ****p ≤ 0.0001.

Finally, we wanted to determine if *NTHI* NRel as generated by either mechanical disruption or co-incubation with P11 exhibited similar gene expression profiles to those generated via the action of the anti-DNABII monoclonal antibody (Mokrzan et al., 2020). We have previously shown a strong similarity between genetic markers of NRel cells and lag phase bacteria. To test this in our cells released from mechanical and P11 disrupted biofilms, we used qRT-PCR to examine the relative expression of *fis*, a signature and predominant gene canonically associated with lag phase of bacterial growth (Rolfe et al., 2012). Expression was significantly greater in the three populations of NRel tested compared to planktonic *NTHI* (Figure 4A). Interestingly, the different methods of generating the NRel cells demonstrated significant differences from each other, with the mechanical disruption resulting in significantly greater *fis* expression (∼400-fold) than the anti-DNABII treated cells (over 200-fold), which themselves were significantly greater than the cells released from P11 disrupted biofilms (almost 100-fold). Despite these metabolic differences however, collectively these results suggest that all three methods release NRel from biofilm residence into a phenotypic state that appears to mimic lag phase.

Changes to multiple processes, including drug uptake, efflux, and the direct mechanism of an antibiotic, can result in sensitivity to antibiotic killing in bacteria (Kapoor et al., 2017). To compare our disruption methods against our previous results and verify the possible mechanisms for the enhanced antibiotic sensitivities, we also examined the relative gene expression of *folP* and *acrR* via qRT-PCR, two additional targeted genes that are canonically associated with lag phase and that we have shown are upregulated in NRel induced by the action of anti-DNABII (Mokrzan et al., 2020). *folP*, which encodes the protein target for sulfamethoxazole (Huovinen, 2001), was significantly increased in each NRel population compared to planktonic *NTHI* (Figure 4B). Whereas both the mechanical and anti-DNABII disrupted NRel showed significantly greater *folP* expression than that of the P11 NRel, they were not significantly different from each other.

β-lactam antibiotics can be transported via the ArcAB efflux pump, which is controlled by the transcriptional repressor ArcR (Anes et al., 2015). Enhanced expression of which, we argue, would result in the increased sensitivity of *NTHI* to A/C (Mokrzan et al., 2020). Relative expression of *acrR* was significantly elevated in all three populations of NRel (Figure 4C). While their *acrR* expression profiles were not significantly different from each other, they did trend toward the pattern seen in the other tested lag phase genes. Collectively, these patterns of relative gene expression support the observed enhanced sensitivities to antibiotic mediated killing, which is consistent with the NRel phenotype.

## 4 Discussion

The global rise of antimicrobial resistance (AMR), along with the recalcitrant and recurrent nature of biofilm-related diseases, warrant the need for new technologies to combat them. While many such strategies are currently in different stages of development and testing, the predominant option of treatment remains with antibiotics that are growing increasingly ineffective. Thus, inhibiting biofilm formation and/or disrupting biofilm-resident organisms to extract them from the protective shells of their EPS matrices is of paramount importance to clearing these infections or ideally, preventing them altogether.

In previous studies, we have shown that a monoclonal antibody that targets the DNABII family of bacterial DNA-binding proteins is highly effective at driving the collapse of biofilms consisting of single or multiple species of pathogenic bacteria, and releasing them into a transient, yet highly vulnerable state. Indeed, regardless of the specific mode of release, multiple laboratories have shown increased sensitivity to antibiotics once the resident bacteria are free of the biofilm (Marks et al., 2013; Zemke et al., 2014, 2020; Chambers et al., 2017; Fleming et al., 2017; Howlin et al., 2017; Fleming and Rumbaugh, 2018; Goodwine et al., 2019; Redman et al., 2021; Chao et al., 2015; Chua et al., 2014; Kalia and Sauer, 2024; Kaplan et al., 2012). Marks et al. (2013) triggered *S. pneumoniae* biofilm dispersion by interkingdom signaling induced by virus infection, with significant dispersal seen within 10–30 min, and a resulting antibiotic susceptibility lasting for at least 2 h. Chua et al. (2014) showed that *P. aeruginosa* biofilms could be dispersed within minutes by reduction in cyclic-di-GMP levels with increased antibiotic susceptibility also lasting 2 h. In Chambers et al. (2017), *P. aeruginosa* biofilms were triggered to disperse by cues such as nitric oxide and glutamate. Dispersion was detected within 15 min after induction with varying durations of increased antibiotic susceptibility based on the trigger and antibiotics used (Chambers et al., 2017). Kaplan et al. (2012), Whitchurch et al. (2002) were able to disrupt biofilms of staphylococci and *P. aeruginosa*, respectively, in as little as 2 min of treatment with DNase I, although they did not specifically look at the duration of increased antibiotic susceptibility. Other methods that induce this newly released phenotype of enhanced susceptibility include depletion of pyruvate availability (Goodwine et al., 2019), inability to aerobically respire (Zemke et al., 2014), and the use of disruptive nanoparticles (Gupta et al., 2017), among others. While the methods differ and offer varying windows of increased antibiotic susceptibility, fast bacterial biofilm disruption methods share the ability to confer the increased antibiotic sensitivity phenotype.

In this NRel state, bacteria are highly susceptible to MIC and even sub-MIC antibiotic concentrations previously measured for the planktonic state (Kurbatfinski et al., 2022, 2023), as well as host immune clearance (Wilbanks et al., 2023). NRel also exhibit a unique gene expression profile, such as upregulation of genes canonically associated with lag phase, outer membrane porins and multi-drug efflux pump repressors, as well as down-regulation in genes involved in protective functions e.g., mitigation of oxidative stress (Wilbanks et al., 2023).

Herein, we have shown that bacterial cells that resulted from our rapid biofilm disruption methodologies displayed the same three hallmarks of the NRel phenotype, particularly as demonstrated by *NTHI*. The mechanical disruption methods used were able to induce 1.5- to 2-fold increased antibiotic sensitivity in *NTHI*, *MRSA*, *P. aeruginosa*, and *S. pneumoniae*, similar to the 1.5- to 2.5-fold increase to antibiotic sensitivity seen in our previous anti-DNABII method of biofilm disruption (Kurbatfinski et al., 2022, 2023), while the P11 method of *NTHI* biofilm disruption was able to induce a 3- to 4-fold increase in antibiotic sensitivity. Although the possibility remains that this increase in antibiotic sensitivity could also reflect sublethal injuries to the cells, such as membrane damage, altered membrane permeability, or oxidative stress effects, the uniform disruption methods across both planktonic and NRel cells control for potential sublethal damage, likely reflecting an inherent biological difference rather than a procedural artifact. Furthermore, this increased antibiotic susceptibility occurred in a time-dependent manner in *NTHI*, consistent with the NRel phenotype as seen in our anti-DNABII rapid release approach (Wilbanks et al., 2023). While time to reach a significant increase in relative antibiotic killing of NRel compared to planktonic *NTHI* differed between each method, significant differences were observed in NRel populations induced by each method between 1 and 4 h, with peak differences generally at 2 h post-disruption, before trending back to near-planktonic levels within 6 h. Earlier work has also shown that anti-DNABII induced *NTHI* NRel displayed preferential killing by a β-lactam antibiotic than by a sulfonamide (Wilbanks et al., 2023), consistent with our current results, albeit to different extents. Whereas the cells released from anti-DNABII disrupted biofilms did not reach a significant increase in antibiotic sensitivity to T/S, the cells released from mechanically disrupted biofilms reached significance at 2 h and the cells released from P11 disrupted biofilms reached significance from 1 to 4 h. Still, it is remarkable that these different experimental designs show such similar kinetic profiles.

Rapidly disrupted *NTHI* also exhibited similar significant gene expression profiles in the specific genes tested compared to planktonic cells. While the upregulation of *fis*, *folP*, and *acrR* is consistent with prior observations of a lag-phase-like signature, future work will include a thorough analysis of this broader physiological state. Although the methods used displayed significant or trending differences from each other, the duration of treatment prior to the collection of NRel for RNA isolation could help to explain the differences seen in relative gene expression. We acknowledge that the 15-min incubation required for the preparation of RNA (the shortest period wherein multiple samples could be prepared in parallel with the lowest precision error) could affect the desired instantaneous transcriptional state of the bacteria. It may be that the isolated RNA captured in part an early adaptive state rather than the instantaneous NRel transcription, an unavoidable conceit in our experimental results. That said, our data in Figure 3 shows that antibiotic sensitivities increase with time for the first few hours after bacteria are released from the biofilm regardless of the NRel technique. Thus, although it is possible we have failed to identify a new window of antibiotic sensitivities prior to 15 min, we think this unlikely. While different disruption rates or the extent of biofilm disruption, i. e., producing single cells as opposed to different magnitudes of aggregates, could explain the differences seen between the methods examined here and previously, the fact that there are differences means that while all these quickly released formerly biofilm-resident bacteria have similar NRel phenotypes, they are not likely identical. While the NRel are clearly bigger than planktonic cells but much smaller than the original biofilm, it also remains unclear to what degree the EPS was affected. Future work will include assessing any changes to the quantity and composition of the EPS in each NRel state as well as directly testing the hypothesis that the properties of NRel bacteria are in part dependent on the method from which they are derived.

Many aspects of this vulnerable NRel state of *NTHI* have been elucidated in our previous studies (Mokrzan et al., 2018, 2020; Kurbatfinski et al., 2022, 2023; Wilbanks et al., 2023). The bacteria newly released from biofilms appear to resemble bacteria in lag phase, as observed by others (Chua et al., 2014), and similarly display significant up- or downregulation of specifically profiled genes. In *P. aeruginosa* freshly dispersed via active programmed release, genes associated with type IV pili, pyoverdine, type III and IV secretion systems and antibiotic resistance are down-regulated, while genes involved in swimming motility, Hxc type II secretion systems, various virulence factors and metabolic and energy-generating systems are up-regulated (Kalia and Sauer, 2024). Whereas there is often increased virulence reported in freshly dispersed cells (Kalia and Sauer, 2024; Chua et al., 2014), this is not a phenomenon observed with rapidly-disrupted NRel (Jurcisek et al., 2025). In contrast, when rapidly released from biofilm residence by physical disruption, as opposed to programmed dispersal, we believe that bacteria are in a state where they are initially poorly equipped to handle the deleterious effects of antibiotics or innate immune effectors. This may be explained by the fact that bacteria resident within a biofilm are often metabolically less active (Uruén et al., 2020). Though these defensive abilities are eventually regained through adequate gene expression, the NRel phenotype nonetheless provides an opportunity for more effective eradication of the formerly biofilm-resident bacteria.

The biofilm matrix is maintained by the interaction of various macromolecules. Chief among these is eDNA. Due to the polyanionic nature of eDNA, positively charged molecules are critical in the maintenance of its structure and structural integrity. Indeed, we have discovered that the DNABII family of basic DNA binding proteins fulfills the aforementioned role (Guéroult et al., 2012; Devaraj et al., 2018). Targeted removal of DNABII proteins collapses the preformed biofilms (Devaraj et al., 2015; Gunn et al., 2016; Novotny et al., 2013; Rocco et al., 2018) with concomitant susceptibility of released bacteria to both antibiotics and innate immune effectors. Moreover, other methods of positively charged molecule depletion e.g., depletion of divalent cations from biofilms using EDTA also creates a sensitizing effect on *NTHI* biofilm (Cavaliere et al., 2014). Our new data reported here are consistent with the notion that depletion of positively charged molecules via the P11 cation exchanger resin was sufficient to rapidly undermine biofilms to generate released cell populations that are in the NRel state. While it is tempting to assume that the DNABII proteins are likely the primary species sequestered by the P11 resin, this idea is largely inferential as it remains to be seen if they are the only positively charged molecules that upon depletion elicit disruption of biofilms. Although it is beyond the scope of this manuscript, future work will include the characterization of the specific mechanisms of P11 biofilm disruption. While the physiochemical properties of P11 may limit direct clinical suitability, it clearly demonstrates proof-of-principle efficacy.

The noted similarities between rapid, physical means of disruption and reagent-based methods indicate that the production of the NRel state is consistent with a rate-dependent model, and we can likely induce the NRel phenotype via a rapid, physical approach independent of the means to accomplish disruption. As such, we posit that the relative speed of the disruption method matters, in that quickly releasing bacteria promotes the greatest shock to the cells, leaving them the most vulnerable and ill-equipped to deal with standard treatment options and effectors of the innate immune system. Per our hypothesis, slower methods of bacterial release would likely allow the bacteria time to adapt and equip themselves with the necessary means to survive outside of the protection of a biofilm. As demonstrated by the NRel phenotype’s transient nature, there is a time limit before the bacteria adapt back to their free-living, planktonic state of growth. Missing that window of opportunity to eradicate these highly vulnerable cells would make treatment that much more difficult.

One major limitation of the approaches used herein as a therapeutic method is that it is often not feasible to physically access the sites of internal infections, thereby making mechanical disruption of these biofilms extremely difficult. Although this study provides proof-of-concept to further our understanding of this unique NRel phenotype, there are opportunities for the usage of rapid, physical means of disruption in clinical practice. Mechanical disruption using jets of water have been developed and used for the removal of pathogenic biofilms, such as irrigation and debridement of surgical site infections and indwelling devices (Koo et al., 2017). Although these jets can remove a significant amount of biofilm from the area, there is the possibility of the released bacteria re-seeding and spreading across the site surface within the water itself to re-establish infection at the same or different location. Indeed, it is also unlikely that biofilm disruption by water jets would break up the bacteria into small enough aggregates or even single cells, necessitating further fine tuning to these procedures to achieve an NRel state. This obstacle likely contributes to the low success rate of irrigation and debridement alone in treating periprosthetic infections (Fabbri et al., 2016). Most approaches to biofilm-specific therapies in these situations still consist of conventional antibiotics or topical antimicrobials (Koo et al., 2017). However, one could envision the addition of antimicrobial agents to more advanced water-based jets so that as they create the necessary mechanical forces to disrupt the biofilm, the fluid simultaneously acts as a delivery device for an appropriate antimicrobial.

Nonetheless, we continue to investigate and support our approach to treating bacterial biofilm diseases, wherein our novel anti-DNABII antibody is administered in a therapeutic regimen, with or without the simultaneous co-administration of an effective antibiotic. Taken together, beyond our further understanding of the NRel phenotype, our results are significant in that they shift the conversation from molecule-specific effects toward physiological state transitions and suggest that fast acting treatments that disrupt biofilms, rather than trigger programmed dispersal, whether reagent-based or otherwise, are likely to induce a similar NRel state.

## Data Availability

The raw data supporting the conclusions of this article will be made available by the authors, without undue reservation.
