# Supplementary material for: Rapid means of biofilm disruption induce the newly released (NRel) phenotype of enhanced antibiotic sensitivity
Source: Front Microbiol. 2026 Apr 10;17:1734540. doi: 10.3389/fmicb.2026.1734540 (PMC13106152; doi:10.3389/fmicb.2026.1734540)
Supplement: Supplementary file 1 [file Table_1.docx]

**Rapid Means of Biofilm Disruption Induce the Newly Released (NRel) Phenotype of Enhanced Antibiotic Sensitivity**

**Supplementary Information**

| **Supplemental Table 1. Primers used in this study** | |
| --- | --- |
| **Primer** | **Sequence** |
| acrR-forward | CGGCGATAAATTTAGCCTCTGA |
| acrR-reverse | TGAATCGCACGCCAAGAG |
| fis-forward | TAATCCTGCCGATGCCTTAAC |
| fis-reverse | CGGGTTTGATTACCACGAGTAT |
| folP-forward | TGCTGGATTTCTGTCGATTCTT |
| folP-reverse | GCTCTTGCAAAGCACGAATATC |
| 16S-forward | AAAGGAGACTGCCAGTGATAAA |
| 16S-reverse | CCCTCTGTATACGCCATTGTAG |


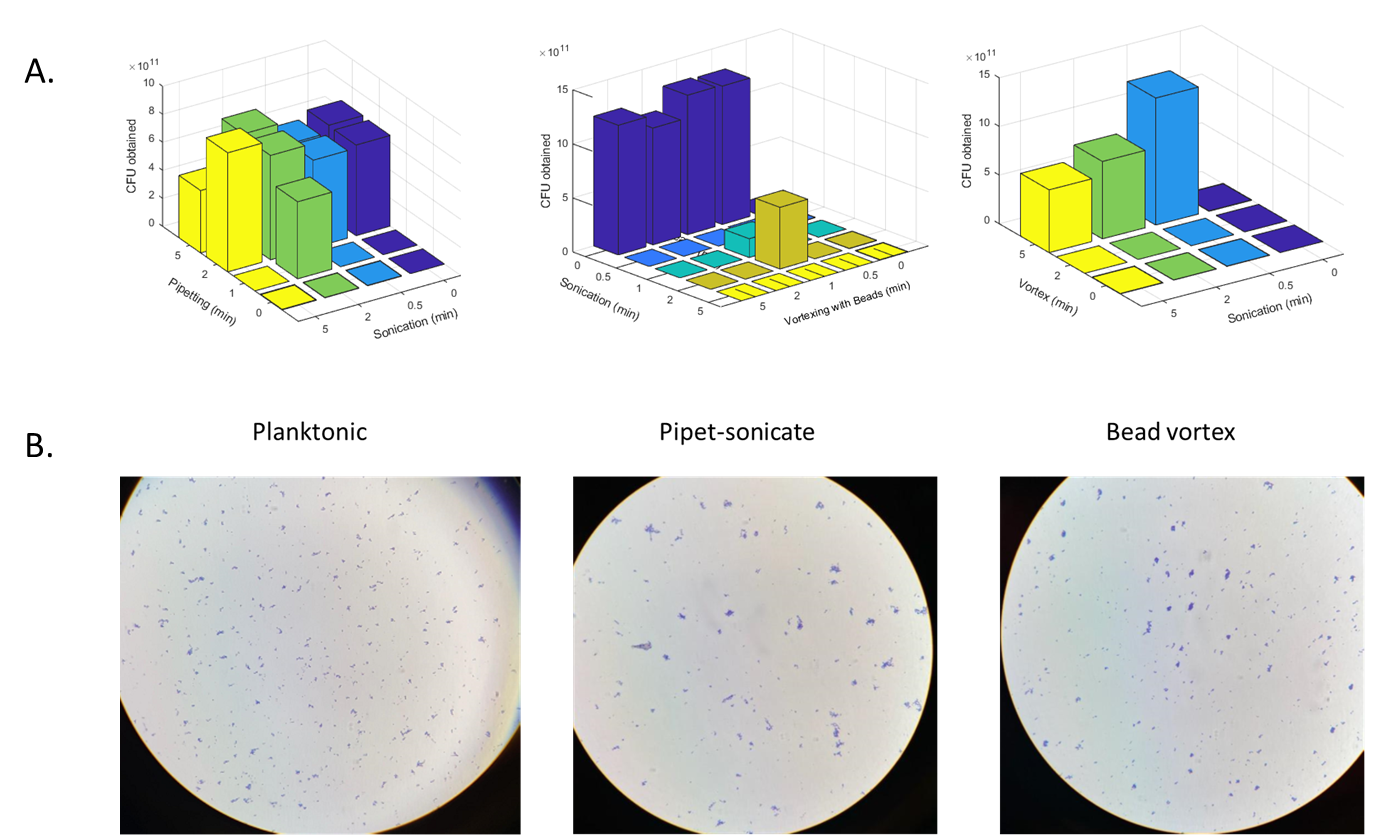


**Supplemental Figure 1** - **Mechanical Biofilm Disruption Methods** (A) Initial screening results comparing different combinations of mechanical disruption methods used in attempts to generate the greatest number of viable NRel populations (B) Crystal Violet stain of planktonic NTHI (left) compared to 16hr NTHI biofilms disrupted with either 2 min of pipetting with 2 min of water bath sonication (middle) or 2 min of vortexing with glass beads (right).

**
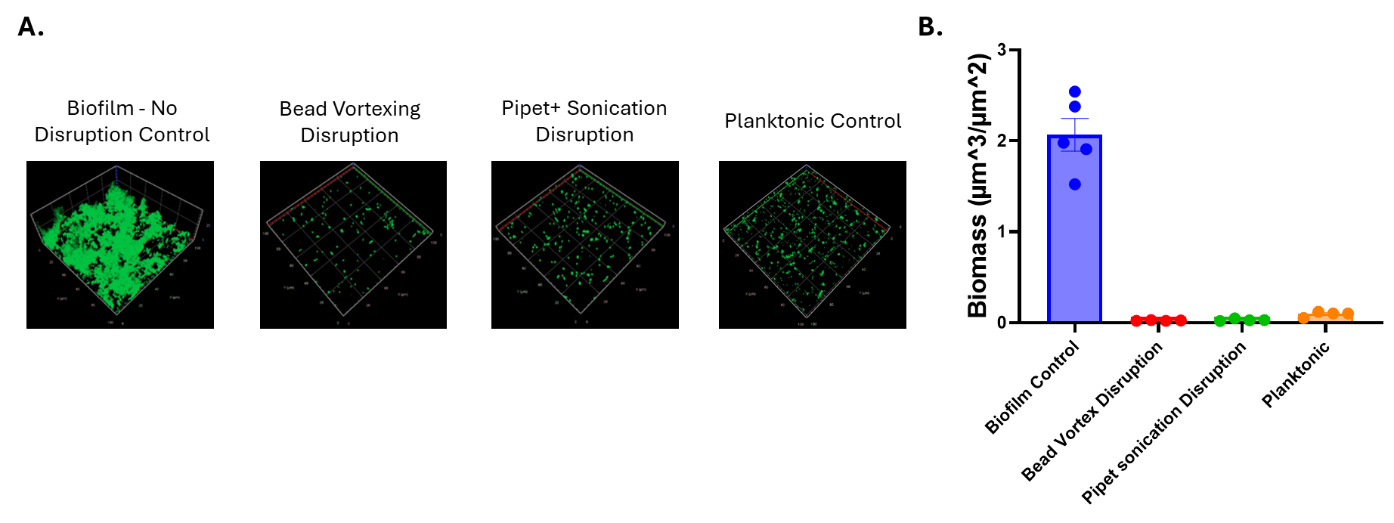
**

**Supplemental Figure 2** - **Mechanical Biofilm Disruption significantly reduces biofilm biomass.**  Disruption of biofilm represented by the reduction in biomass. NTHI biofilms were allowed to grow in an 8 well chamber slide for 16 hours. Biofilms were removed by pipetting and disrupted either with the pipetting + sonication or the vortexing with glass beads methods of mechanical disruption. NRel formed from both methods were added back to the chamber slide wells (in addition to planktonic cells) and incubated for 5 minutes. The biofilms were stained with Live/Dead stain and visualized using CLSM. (A) Representative CLSM images are shown for the non-disrupted control, bead vortex disruption, pipet sonication disruption and planktonic cells. (B) The average biomass was calculated using COMSTAT software and plotted. The data are presented as mean ± SEM.

**
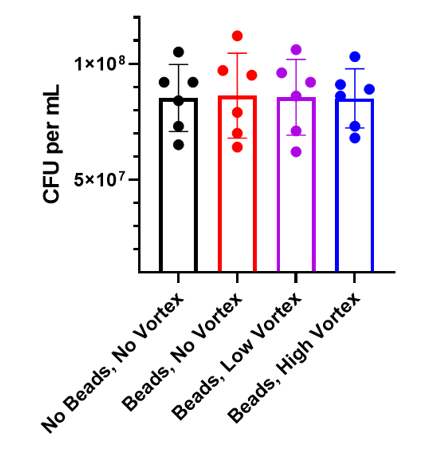
**

**Supplemental Figure 3 – Mechanical disruption by bead vortexing does not affect cell viability.** To obtain planktonic cultures, 6 mL of sBHI was seeded with approximately 6 individual colonies of freshly plated *NTHI* to achieve an OD_490nm_ of 0.1, and allowed to grow at 37 °C, 5% CO_2_ until mid-log phase (2-3 hours) prior to being aliquoted into Eppendorf tubes with or without beads. The cell suspensions were then subjected to no vortexing, vortexing at setting 3 (low speed), or vortexing at setting 10 (high speed and the speed used throughout the manuscript for this disruption method) on a Fisher analog vortex. Concentrations were determined via serial dilution of the resulting cell suspensions and plating for CFUs. As shown in the figure, the presence or absence of beads did not affect viable cell counts, with the speed of vortexing with the beads likewise demonstrating no effect on cell viability.
